# Discovering Stick-Slip-Resistant Servo Control Algorithm Using Genetic Programming

**DOI:** 10.3390/s22010383

**Published:** 2022-01-05

**Authors:** Andrzej Bożek

**Affiliations:** Department of Computer and Control Engineering, Rzeszow University of Technology, al. Powstańców Warszawy 12, 35-959 Rzeszów, Poland; abozek@prz.edu.pl

**Keywords:** servo control, stick-slip effect, sensor feedback, genetic programming

## Abstract

The stick-slip is one of negative phenomena caused by friction in servo systems. It is a consequence of complicated nonlinear friction characteristics, especially the so-called Stribeck effect. Much research has been done on control algorithms suppressing the stick-slip, but no simple solution has been found. In this work, a new approach is proposed based on genetic programming. The genetic programming is a machine learning technique constructing symbolic representation of programs or expressions by evolutionary process. In this way, the servo control algorithm optimally suppressing the stick-slip is discovered. The GP training is conducted on a simulated servo system, as the experiments would last too long in real-time. The feedback for the control algorithm is based on the sensors of position, velocity and acceleration. Variants with full and reduced sensor sets are considered. Ideal and quantized position measurements are also analyzed. The results reveal that the genetic programming can successfully discover a control algorithm effectively suppressing the stick-slip. However, it is not an easy task and relatively large size of population and a big number of generations are required. Real measurement results in worse control quality. Acceleration feedback has no apparent impact on the algorithms performance, while velocity feedback is important.

## 1. Introduction

**Figure 1:**
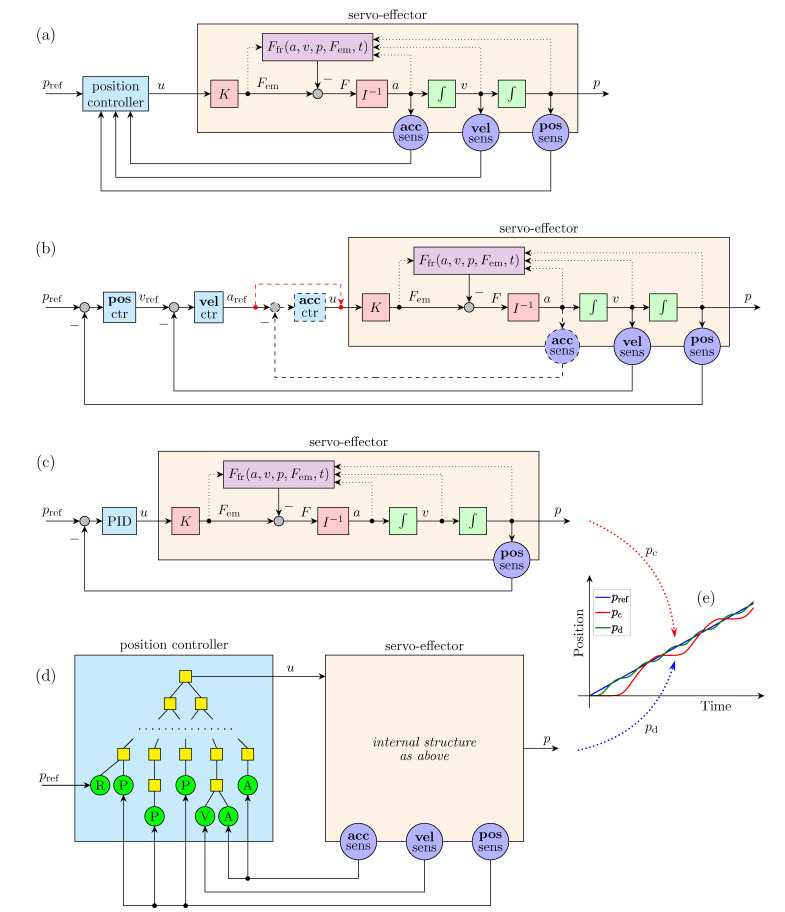
Servomechanizm with friction: (**a**) general control structure; (**b**) cascade control structure; (**c**) single loop structure with PID; (**d**) stick-slip-resistant structure with parse-tree-based controller; (**e**) stick-slip effect.

**Figure 3:**
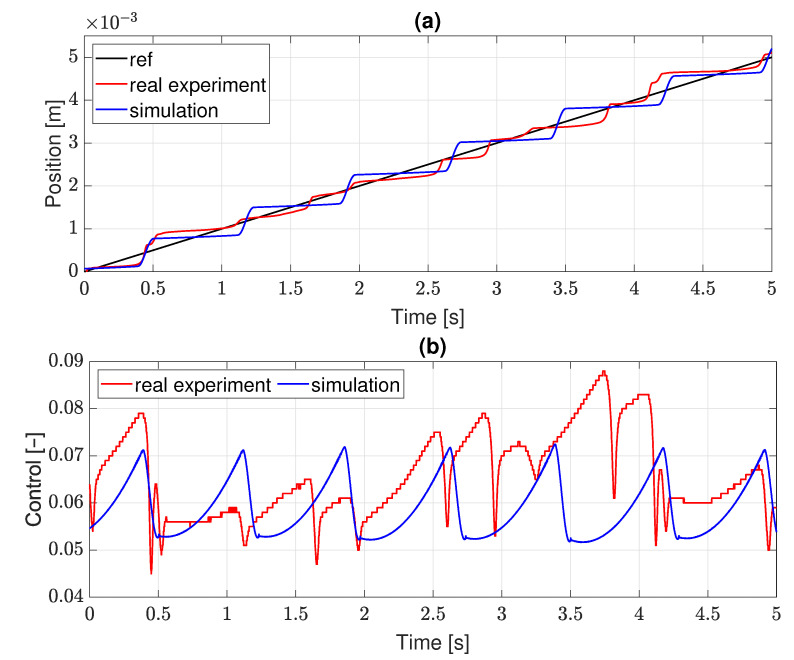
Stick-slip effect in real and simulated servo system: (**a**) position; (**b**) control.

**Figure 4:**
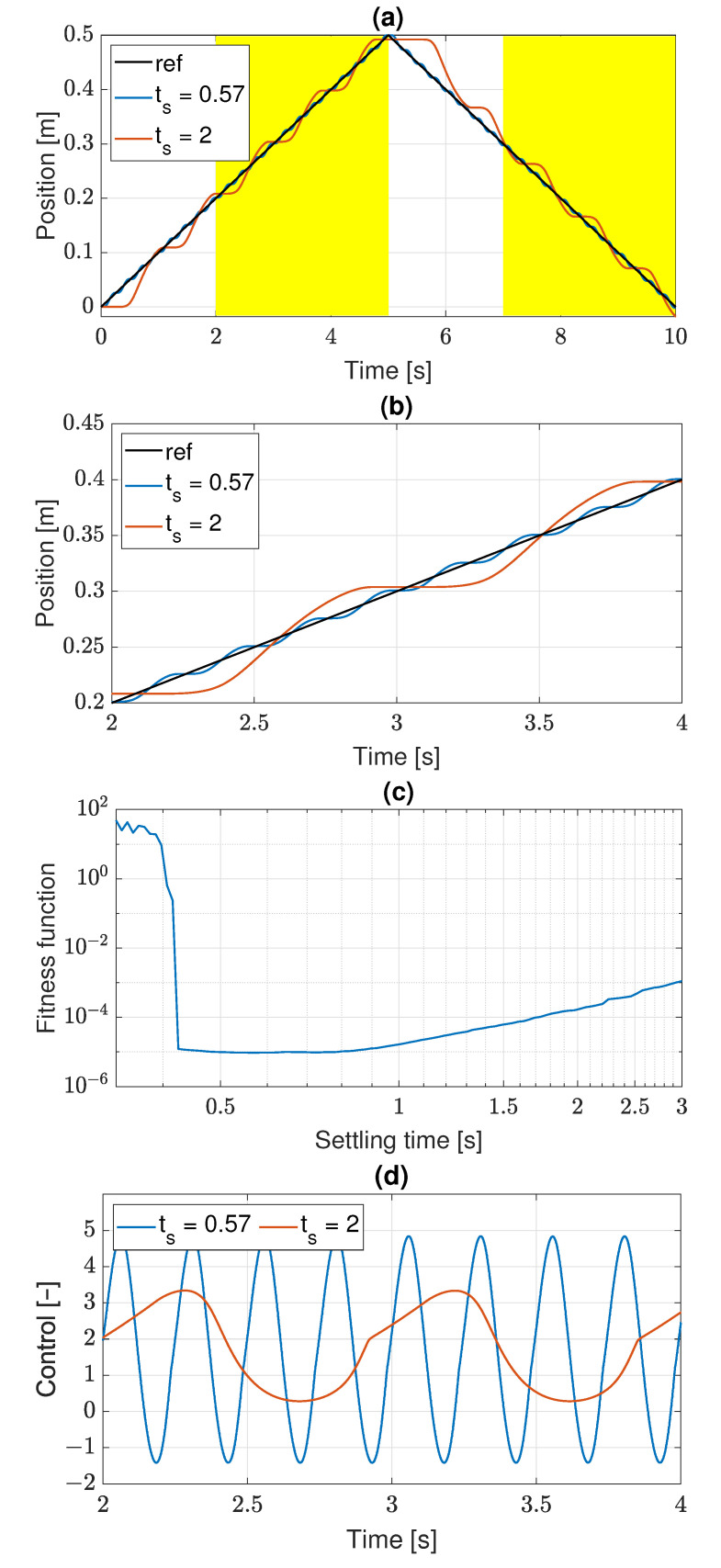
Learning trajectory and PID control: (**a**) reference position and stick-slip effect; (**b**) detailed view of stick-slip effect; (**c**) fitness function sensitivity on settling time; (**d**) control signal.

Friction is a complicated nonlinear dynamic phenomenon. In mechatronics and robotics, it is a force disturbing control processes of mechanical motion. In particular, friction causes tracking errors of position control devices, commonly called as servomechanisms, or servos for short. Stick-slip effect is a specific type of such errors that emerges while a servo moves continuously with a relatively low speed. It has a form of cyclic oscillations around (or above/below) a reference trajectory (see examples in Figure 1e, Figure 3 and Figure 4a,b).

In this work, an original paradigm and tool for design of a control algorithm for stick-slip compensated servo systems are proposed. The paradigm assumes the use of a machine learning approach to directly discover an effective control algorithm. The paradigm has been verified in simulation experiments, and its usefulness has been proven. Genetic programming (GP) [1] has been chosen as the machine learning tool. It is very well suited for discovering control algorithms because the GP constructs individuals that can be decoded as programs. In the implemented learning framework, GP nodes have been defined that represent input data, arithmetic operations, nonlinear functions, as well as unit delays. This makes the genetic process possible to generate a broad range of control algorithms.

The stick-slip has been isolated to deal with a well defined problem in this work. Besides a smooth motion during which the stick-slip emerges, a servo can stabilize a constant reference position or execute a transient motion in reaction on a reference trajectory step. These tasks are quite different; in particular, step responses are less susceptible to friction and standard linear or optimal control approaches may be sufficient for them. For that reason, generalization of the GP learning task to deal with any type of reference trajectory at once is not advantageous, as the discovered algorithm could not specialize enough in any kind of disturbance.

A genetically obtained control algorithm can be considered in some sense to be an intelligently discovered program processing sensor data. Special attention has been paid to sensors in the modeled servo system. The learning efficiency has been tested under the assumption that the discovered control algorithm has full access to measurement of position, velocity, and acceleration, as well as for the configurations in which acceleration, and optionally also velocity, are inaccessible. The scenarios of ideal and real measurement have also been compared.

The design of a servo system suppressing friction disturbances combines several aspects, mainly: friction modeling, identification of friction in real systems, implementation of friction compensators and compensated controllers, selection of appropriate sensors. The up-to-date review of friction modeling in servo machines [2] reveals that it is a very complex issue. The correct friction model and its compensation can significantly improve servo tracking accuracy, especially at low motion velocity. One of the first friction models was proposed by Dahl [3]. However, this model does not capture the Stribeck effect [4,5], which is crucial for low-speed motion servo disturbances [6], especially the stick-slip oscillations. The Stribeck effect is taken into account in the well-known LuGre model [7,8]. This model has many variants and extensions. In particular, the variant proposed by de Wit et al. [9] has been used in this work, as it captures the main friction components important for modeling of servo disturbances, but it remains simple for numerical implementation and fast in simulation. Having a parametric friction model, the common approach is to identify its parameters using an optimization technique. Many classic and intelligent methods are used: distinguishing inertia torque and friction torque [10], iterative minimisation of the error between a model and experiment result [11], gradient-based convex optimization [12], genetic algorithms [13], accelerated evolutionary programming [6], and particle swarm optimization hybridized with neural dynamic programming [14]. Approaches involving online adaptative estimation of the motor parameters including friction model coefficients [15] exist. The vast majority of the methods use a time function of servo position, but frequency response analysis is also employed [16]. Similar optimization techniques are applied for tuning servo controllers, e.g., linear quadratic regulator [17] and different variants of genetic algorithms [18,19]. Sometimes, the same method is used for the friction model identification and controller tuning, e.g., glowworm swarm optimization [8], genetic algorithm, and differential evolution [13]. Asymptotic tracking control for nonaffine systems with disturbances [20] is an advanced method proposed for rejection of unknown disturbances. The experiment involving SCARA manipulator confirmed that this approach satisfies design requirements and outperforms a PD controller. In some aspects, the formulation of this control problem and the form of its verification are similar to those from this work, but they also differ in details, e.g., objective definition. It is worth confronting both the approaches in a future comparative study.

While there is probably no published research on the application of the GP for processing sensor data involving servos, this approach has been studied in the general field of sensor technology. A GP-based design of soft-sensors for biochemical systems was proposed, and it was proven that this approach outperforms solutions based on neural network and support vector regression [21]. A GP-based method of data analysis from Compact Airborne Spectrographic Imager hyperspectral sensor was developed that outperformed multiple regression, tree-based modeling, and genetic algorithm partial least squares [22]. The GP approach was successfully employed for recognition of coffee crops on images captured by a satellite [23].

The GP is also used in mechatronics, but not so often [24,25,26]. It may be a result of getting used to parametric models of mechatronic systems. Such models include numerical parameters which values are determined by other optimization and learning methods.

The general structure of the servo position control system considered in this work is presented in Figure 1a. It consists of a controlled servo-effector and a position controller. The controller executes an algorithm calculating the control signal *u* on the basis of the reference position $p_{\mathrm{ref}}$ and feedback signals from sensors. In a general case, three kinematic signals (position, velocity, and acceleration) can be measured and provided to the controller. The jerk feedback [17] is omitted as very rare. The goal of the position control is to minimize static and dynamic errors between the reference position $p_{\mathrm{ref}}$ and the actual position *p* of the servo-effector. It is assumed that the servo-effector includes an electronic device, called as an amplifier or drive, which transforms the control signal *u* into current, as well as an electric motor generating electromagnetic force $F_{\mathrm{em}}=uK$ proportional to the current and to the control signal. The electromagnetic force plays the role of the mechanical force or torque in the case of linear or rotary motor, respectively. The force divided by an inertia parameter *I* (mass or moment of inertia) results in the acceleration *a*. The velocity *v* and position *p* of the servo-effector are consecutive time integrals of the acceleration. The friction force $F_{\mathrm{fr}}$ is also included in the model. It is always directed against the velocity and reduces the effect of the electromagnetic force. In general, the friction force may depend on actually every variables: *a*, *v*, *p*, $F_{\mathrm{em}}$. The structure of the proposed servo-effector model is similar to that used in [15], which turned out to be sufficient for an adaptive algorithm compensating friction in servo control.

The servo controller is often implemented in the nested form presented in Figure 1b. It is split into separate units: an (actual) position controller, velocity controller, and acceleration controller. Each unit uses an input error signal obtained by negative feedback from a proper sensor. In practice, the acceleration feedback and acceleration controller are used rarely [17,27,28,29] and, without them, the output of the velocity controller becomes the control signal. In the majority of industrial servo systems, simple linear controllers: P (proportional) and PI (proportional-integral) are used as the position and velocity controller, respectively [17,30]. The servo control structure can be even simpler after replacement of the nested P-PI controller by a single-loop PID controller, as shown in Figure 1c. In the latter case, the position feedback signal from one sensor suffices for effective control. However, there are limitations of performance of the simple linear servo control. In particular, such a linear control cannot efficiently suppress an impact of nonlinear phenomena, especially the friction force. The stick-slip effect appears when a servo tracks a ramp reference position with velocity in a specific range. Then, instead of smooth motion, the servo position changes according to a stairs-like trajectory with an oscillatory position error, as presented in Figure 1e.

The approach introduced in this work to suppress the stick-slip effect is illustrated in Figure 1d. It assumes a return to the general structure from Figure 1a, i.e., all sensor signals are again provided to one common control algorithm. However, the algorithm is discovered by the GP. It should make it possible to find an algorithm outperforming the simple PID in the stick-slip suppression. This research concept has been comprehensively verified in the work. GP learning experiments have been conducted on a simulated servo system with friction, as a real-time realization will last an unacceptably long time.

The proposed approach of discovering of a servo control algorithm is novel, and no similar solution is known to the author. There are a few aspects of originality. Genetic programming as a machine learning technique has not been used for such a nonlinear control problem like stick-slip suppression, neither in the context of servo control nor processing multi-sensor data. This method is powerful and convenient because no a priori knowledge about a controlled process is required; instead, the learning is driven by a fitness function. As there is no free lunch, the flexibility comes at the cost of long training time. The research has confirmed these supposed properties that GP finds control algorithms of excellent performance, but it takes a long time, such that the learning is too slow for real-time execution, and it needs a simulated system. There are also more detailed research results, e.g., the acceleration feedback seems not to increase the control quality, while the velocity feedback is important, both for the sensors modeled as ideal and real. Besides the main results, the proposed design of a genetically constructed controller exemplifies a concept of *dynamic genetic program*, used perhaps for the first time in this work. This is a genetic program in which memory cells representing discrete-time state variables are employed as node functions (unit delays), making it possible to encode complex dynamical systems.

## 2. Materials and Methods

### 2.1. Structure of an Experimental Simulation System

**Figure 2:**
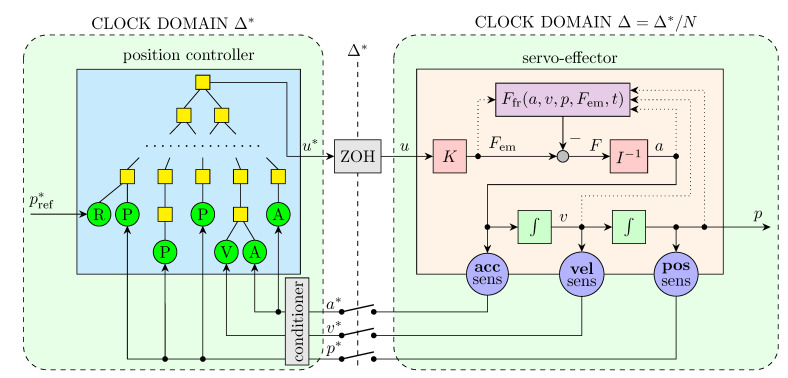
GP-based learning system.

The structure of the experimental simulation learning system is shown in Figure 2. It is a specialization of the concept introduced in Figure 1d. A control algorithm discovered by the GP is dedicated for a digital controller; hence, it has to be executed with a given cycle time. This is ensured by the clock domain $\Delta^{*}$, which imposes that values of all the signals inside it, namely $p_{\mathrm{ref}}^{*}$, $u^{*}$, $p^{*}$, $v^{*}$, $a^{*}$, are recalculated cyclically every time interval $\Delta^{*}$. The servo-effector is simulated inside a separate clock domain with a shorter cycle time $\Delta =\Delta^{*}/N$ to better emulate continuous-time dynamics expected of this subsystem. The integrators inside the effector are modeled by approximate trapezoidal discrete rule, i.e., the relationship between input $x_{k}$ and output $y_{k}$ signals of an integrator has the form $y_{k}=y_{k-1}+\left(x_{k-1}+x_{k}\right)\Delta /2$. The control signal value $u^{*}$ computed by the position controller is held in the ZOH (*zero-order hold*) block, until computation of its new value after the interval $\Delta^{*}$. The readings from sensors are sampled in every controller cycle. Such a connection between the controller and effector reflects the structure of a real digital servo control system.

The value $\Delta^{*}=1\;\mathrm{ms}$ has been used in the research, which is typical for servo control. The step of the servo-effector simulation is 10 times faster, so N=10 and Δ=0.1ms, providing a compromise between precision and speed of simulation.

### 2.2. Friction Model

In the simulation model of the servo-effector, the friction force $F_{\mathrm{fr}}$ is calculated on the basis of the formula proposed in [9]

(1) $$F_{\mathrm{fr}}^{*}\left(v\right)=\left\{\begin{array}{cc} \pm F_{s}, & v=0,\\ \left(F_{c}+\left(F_{s}-F_{c}\right)e^{-{\left(v/v_{s}\right)}^{2}}+F_{v}\left|v\right|\right)\mathrm{sgn}\left(v\right), & v\neq 0. \end{array}\right.$$

However, the expression isolated for the interval v=0 is difficult for numerical processing due to the zero length of this interval and the ambiguous force sign. For that reason, the extension proposed by Karnopp [31,32] has been used

(2) $$F_{\mathrm{fr}}\left(v\right)=\left\{\begin{array}{cc} \pm F_{\mathrm{ex}}, & |v|<\alpha ,\\ F_{\mathrm{fr}}^{*}\left(v\right), & |v|\geq \alpha , \end{array}\right.$$

where a small enough velocity modulus |v|<α is treated as the actual zero velocity and the friction compensates the external force $\pm F_{\mathrm{ex}}$, otherwise, i.e., for |v|≥α, the basic Formula (2) for the friction force is applied. The used model takes into account a few physical phenomena of friction, namely static, Coulomb, and viscous components, represented by the parameters $F_{s}$, $F_{c}$, and $F_{v}$, respectively. The model also represents the Stribeck effect, related to the Stribeck velocity $v_{s}$.

Prior to implementation of the complete system presented in Figure 2, the model of servo-effector with the friction function described in (1) and (2) was connected to a classic PID controller to verify if it generates the expected stick-slip effect. The result is shown in Figure 3, where an outcome from a real servo system is also provided for comparison. Friction parameters and PID settings of the simulated structure have been adjusted experimentally to get similar stick-slip oscillations in the simulated and real systems in terms of their amplitude and period. The effects are indeed similar, even though the simulation cannot reflect all properties of the physical system, e.g., it does not reveal stochastic behavior, evident for the real servo.

### 2.3. Learning Trajectory and Fitness Function

The learning trajectory is a time function of reference position to be followed by the servo-effector controlled by an algorithm constructed in the GP evolutionary process. It has been defined in the form

(3) $$p_{\mathrm{ref}}^{L}\left(t\right)=\left\{\begin{array}{cc} 0.1t, & t\in [0,5],\\ 1-0.1t, & t\in (5,10]. \end{array}\right.$$

The function plot has a triangular shape shown in Figure 4a. The reference position first grows with the constant slope 0.1, and then returns to zero with the slope −0.1. The ramp shape of the trajectory is necessary to induce a stick-slip effect that is to be reduced by the discovered control algorithm. The learning trajectory has two parts to reduce a possibility of control algorithm overfitting. In the case of a very simple reference trajectory, like in Figure 3, the GP process is likely to discover an algorithm which ignores the actual reference position and generates its representation internally. The time span of the learning trajectory should be as short as possible to speed up the learning process, but it has to be long enough to reveal established stick-slip oscillations. As a compromise, the time of 10 s has been chosen.

The fitness of a control algorithm is measured by similarity between the reference and actual servo trajectory expressed by the mean sum of squared errors (MSE), where the error is a difference between time-related positions on both the trajectories. The smaller (closer to zero) value of such defined fitness function, the genetic individual representing a control algorithm, is better fitted. The intervals at the beginning of the rising and falling ramp parts of the learning trajectory have been excluded from the fitness calculation, to omit the transient dynamic processes related to changes of the trajectory shape and to evaluate only the parts with a steady stick-slip effect. The excluded intervals are [0,2] and (5,7], and the fitness function is calculated in the remaining intervals indicated by yellow color in Figure 4a. The calculation is based on the servo-effector position sampled with the cycle time $\Delta^{*}$. The value $p^{*}\left(k\Delta^{*}\right)$ denotes the position at the *k*-th sample, i.e. at the time $k\Delta^{*}$. Finally, the fitness function obtains the form

(4) $${\mathrm{fit}}_{\mathrm{MSE}}=\frac{\sum_{k=1+2/\Delta^{*}}^{5/\Delta^{*}}{\left(p_{\mathrm{ref}}^{L}\left(k\Delta^{*}\right)-p^{*}\left(k\Delta^{*}\right)\right)}^{2}+\sum_{k=1+7/\Delta^{*}}^{10/\Delta^{*}}{\left(p_{\mathrm{ref}}^{L}\left(k\Delta^{*}\right)-p^{*}\left(k\Delta^{*}\right)\right)}^{2}}{N},$$

where $N=\#\left((2/\Delta^{*},5/\Delta^{*}]\cap N\right)+\#\left((7/\Delta^{*},10/\Delta^{*}]\cap N\right)=6000$ is the number of trajectory points involved in the fitness calculation.

### 2.4. Fitness of the PID Algorithm

Before performing the GP learning experiments, the servo-effector has been tested in connection with the standard PID controller to obtain related value of the fitness function (4), needed later for verification if and how much the GP-discovered algorithm outperforms the PID.

For the PID test and for all GP learning experiments, the following parameters of the servo-effector have been used: K=1, I=1, $F_{s}=3$, $F_{c}=0.5$, $F_{v}=1$, $v_{s}=0.1$, α=0.01. The simple values were arbitrarily assigned to *K* and *I*, and then the parameters of friction were adjusted experimentally to obtain a typical stick-slip effect. It has also been assumed that the control signal has the range [−10,10], so it is trimmed to this interval before being provided to the effector.

The PID controller has been tuned using formulas proposed in [30], which depend on the servo-effector gain *k* and a desired settling time $t_{s}$. We ignore the friction in the PID tuning, then k=K/I=1, and the formulas for settings become the following functions of the settling time:

(5) $$k_{p}=\frac{216}{{t_{s}}^{2}},\;k_{i}=\frac{432}{{t_{s}}^{3}},\;k_{d}=\frac{27}{t_{s}}.$$

A sequence of simulations has been performed for $t_{s}\in \left[0.7,3\right]$ and the obtained values of the fitness in function of $t_{s}$ are presented in Figure 4c. The fitness function obtains minimum value of $9.5\times 10^{-6}$ for $t_{s}=0.57\;s$. It represents the best suppression of the stick-slip effect achieved by the PID. If the settling time increases above 0.57 s, the amplitude of the stick-slip oscillations gradually grows. For instance, in Figure 4a,b, the oscillations for the best suppression (blue color) and for a worse case (red color, $t_{s}=2\;\;s$) are compared. The plot in Figure 4b is just an enlargement of the one from Figure 4a for the time interval [2,4]. While the settling time decreases, the amplitude of the stick-slip oscillations becomes smaller, but the amplitude of control signal oscillations grows, as shown in Figure 4d. If the settling time becomes small enough, the limits on control signal make an effective control impossible and the system loses stability, then the fitness function obtain large values, as shown in Figure 4c. On the whole, the plots in Figure 4a–d depict limitation of the PID control in stick-slip effect suppression.

### 2.5. Implementation and Configuration of the Genetic Programming Process

**Figure 5:**
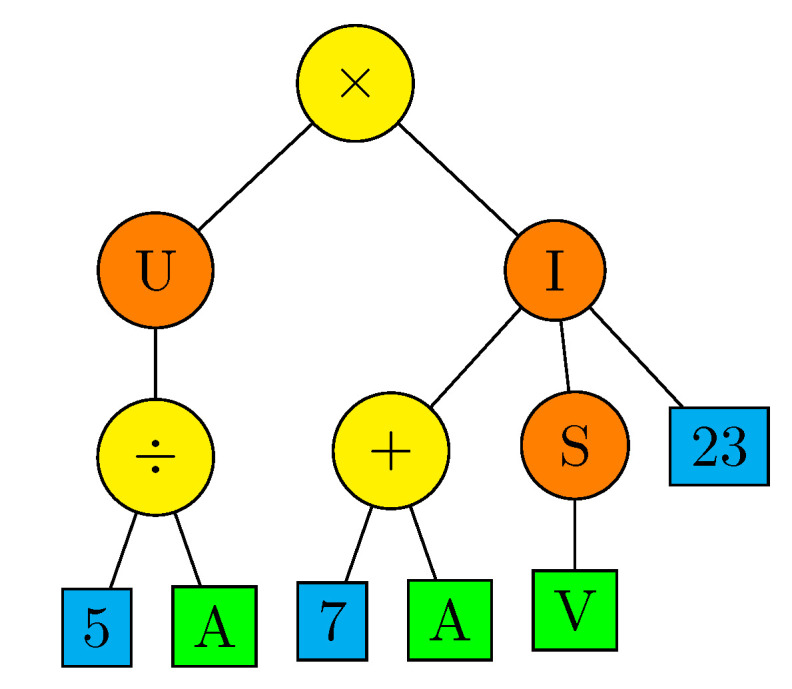
Example of parse-tree.

The ECJ software package [33] has been used for implementation of GP. The ECJ supports “Koza”-style tree-structured GP representation [1]. Genetically discovered programs are encoded by s-expression parse-trees, where leaves (terminals) represent arbitrary data, typically input information or constants, whereas intermediate nodes (non-terminals) are genetic functions or operations which process input data from their children and forward a result to parent nodes. In particular, the root node of such a tree returns a final result of the encoded program. An example of a simple parse-tree is presented in Figure 5. It encodes a program

(6) Prog(A,V):=U(5/A)×I(7+A,S(V),23),

where the nodes “×”, “/”, and “+” execute arithmetic operations, the nodes “U” and “S” represent some unary functions, and “I” represents a ternary function. While it is not necessary for GP in general, the exemplary program processes numerical data, as only such data can be compatible with the arithmetic operators and numerical terminals 5, 7, 23. The terminals A and V should be considered as numerical arguments of the encoded program. The servo control algorithms in the implemented GP system are encoded in a similar way. The reference position and signals from sensors are provided to terminals of an encoding parse-tree and a resultant value obtained in the tree root is used as the control signal for the servo-effector (compare Figure 2).

It is important to define the set of GP node functions properly. One should not omit a function important for the structure of well-fitted programs. Otherwise, the genetic process might substitute such a function with a combination of others, but probability of success will decrease. On the other hand, too large of a set including similar and redundant functions is also problematic, as it may impede constructing of well-fitted individuals due to large combinatorial complexity.

The set of GP node functions used in the research is given in Table 1. Elementary arithmetic operations (addition, subtraction, multiplication, division) form the basis of the set. To avoid “not a number” results, the division has a special safe definition, such that the result is 0 if the divisor is equal to 0. The arithmetic operations suffice for constructing rational expressions over the variables provided to terminals. Two nonlinear node functions are also included: sin and if-else, denoted in parse-trees by S and I, respectively. They increase flexibility of GP-generated programs to express nonlinear relationships. In particular, the sin function may represent periodic behaviors in a control algorithm, possibly useful in the stick-slip suppression, as this phenomenon reveals a periodic form. The if-else expression can model sophisticated decision procedures, piece-wise functions, and so on. The last non-terminal function in the defined set is the unit delay U. It is a special type of node which introduces memory to encoded programs and makes them possible to exhibit dynamic behavior. It works equivalently to a standard $z^{-1}$ discrete-time transfer function. A node with the function U provides to its parent the value obtained from the child in the previous parse-tree evaluation. For this reason, each U node has incorporated a variable saving the previous input. The values of all these variables define current state of the control algorithm, which evolves in consecutive control cycles. Before the first cycle of simulation of an evaluated algorithm, all the state variables are set to 0.

The subset of terminal functions defined in Table 1 includes constant integer values and input signals from the servo-effector. A genetic process can create terminal node with a random integer in the set {1,…,100} and, once the node is present in a parse-tree, the assigned value can be changed to another one (from the same set) in the result of mutation. The set of constants is restricted, due to assumption that the genetic evolution can construct required values combining the integers using arithmetic functions. It is easier to deal with the simple form of constants, e.g., to write them to files without approximation. The terminal functions P, V, A represent current values of the sampled signals from sensors (or related estimators), namely $p^{*}$, $v^{*}$, $a^{*}$, respectively. The terminal R provides the reference position, i.e., the signal $p_{\mathrm{ref}}^{*}$. The terminal C represents the value of control signal $u^{*}$ calculated by an encoded algorithm in its previous evaluation (the previous control cycle). Hence, it is also a form of memory inducing dynamic behavior, but more specific than the function U. It has been introduced because $u^{*}$ is an important signal and the direct access to its previous value may be useful for discovering a well-fitted algorithm.

In addition to design of the GP function set, there are many other parameters to be adjusted in the GP system. The basic configuration used in the research is presented in Table 2. By trial and error, the number of individuals in population and the number of generations were set to 1000 and 500, respectively. This ensures the emergence of good solutions in the genetic evolution. Individuals in the initial population are created randomly using a ramped half-and-half algorithm [33,34] generating parse-trees of depths between 2 and 6. There are many more detailed options, but they have been kept unchanged from the default ECJ settings.

The GP learning experiments were executed in 15 different configurations given in Table 3. For each configuration, the GP learning process was repeated 100 times. The symbols introduced in the table are used to indicate an experiment configuration later in the text. First of all, the experiments have been carried out for different sets of input sensor data. There are options with full data and restricted configurations, i.e., without sensing of acceleration, velocity, both acceleration and velocity, and finally also without feedback of the control signal $u^{*}$. The restrictions are imposed by omitting terminals in the GP function set. This is for the GP to discover which sensors are necessary to effectively suppress the stick-slip. If some sensor is redundant, the result is expected to be not worse after its elimination. It could then be even better, as the evolutionary process has a simplified set of functions to use. In the research, three different levels of mutation have also been compared, namely for the mutation probability equal to 0.1, 0.2, and 0.3. More intensive mutations increase exploring capability of the evolutionary process, but they may destroy well-fitted individuals.

According to the test trajectory definition (3), a real-time experiment evaluating one genetic individual on a physical servo system would take 10 s. One evolutionary process configured according to Table 2 executes almost 500,000 evaluations (reproduced individuals do not need to be reevaluated). Such a learning process needs about two months to complete, and it is to be repeated 100 times for each of 15 variants defined in Table 3. Hence, it is impossible for practical realization, at least using one or only a few physical devices simultaneously. This is a reason why the form of computer simulation has been chosen. The execution of 100 repetitions of the GP learning process for a fixed parameter configuration takes a few hours on a modern personal computer.

## 3. Results

### 3.1. Unconstrained Bang-Bang Control Algorithms

**Figure 6:**
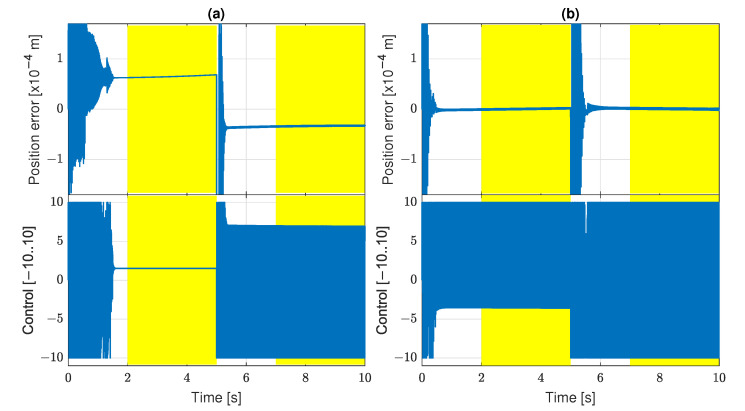
Tracking quality for bang-bang control: (**a**) $J=2.7\times 10^{-9}$; (**b**) $J=1.3\times 10^{-12}$.

The results of experiments performed with the settings described before revealed some problem. The genetic process has a tendency to produce control algorithms which generate an oscillatory bang-bang control signal (Figure 6).

This is quite a predictable result, as the friction force becomes relatively insignificant if the control signal amplitude is maximal. Therefore, the GP system can rather easily discover effective algorithms issuing the bang-bang control. However, such a control is not preferred. First, it may induce mechanical vibrations and overloads in a real system. Second, the high frequency band of this control signal may not be transferred accurately in a real servo structure, and its properties important for stick-slip suppression may be lost.

To prevent the GP system from finding the bang-bang control, a penalty component has been added to the fitness function. During algorithm evaluation, the number *Z* of zero crossings by the signal $u^{*}$ is counted; more precisely, it is the number for which the condition $u^{*}\left(k\Delta^{*}\right)\;u^{*}\left(k\Delta^{*}\;+\;\Delta^{*}\right)<0$ is satisfied. If Z>20, then the fitness function value is increased by a large component, such that the evaluated individual becomes very poorly fitted. Therefore, a few control sign changes are permitted, as they may be unavoidable in the transient phases of servo tracking, but the continuous bang-bang oscillations are banned. All the following results have been obtained in the GP system with this penalty.

### 3.2. Control Algorithms for Servo with Ideal Measurement

We assume first that the measurement is ideal. The value $P_{k}$ provided to parse-tree terminals P in the *k*-th control cycle is equal to the sampled position $p^{*}\left(k\Delta^{*}\right)$ (the conditioner does not change it in any way), which, in turn, is the exact value of the physical signal $p(k\Delta^{*})$ (the position sensor is ideal), the same is true for velocity and acceleration, namely

(7) $$P_{k}=p^{*}\left(k\Delta^{*}\right)=p\left(k\Delta^{*}\right),\;V_{k}=v^{*}\left(k\Delta^{*}\right)=v\left(k\Delta^{*}\right),\;A_{k}=a^{*}\left(k\Delta^{*}\right)=a\left(k\Delta^{*}\right).$$

**Figure 7:**
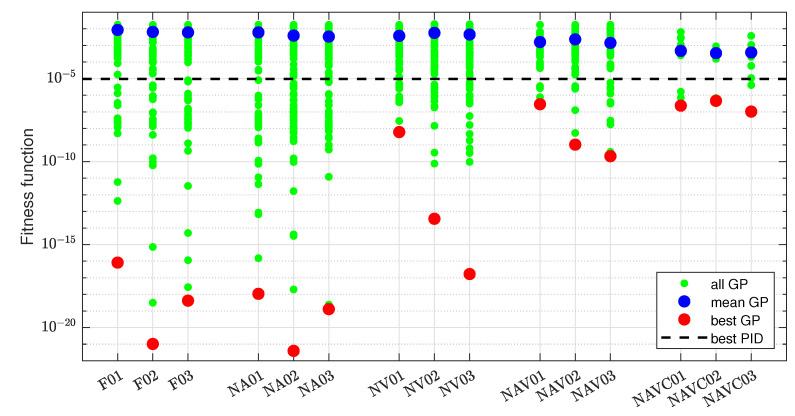
Comparison of GP learning results for ideal sensors.

The overview of the results obtained for the ideal measurement and the configurations indicated in Table 2 is given in Figure 7. The green points represent fitness of the best individuals for each of 100 repetitions of 15 considered configurations. The blue and red points represent the mean and best (minimum) values, respectively, of the fitness function for each configuration. For comparison, the line indicating the best fitness function value obtained by the PID algorithm is also shown. The values of the fitness function span many orders of magnitude, so the logarithmic scale is used.

More details are given in Table 4. The symbol S denotes the set of all best individuals from 100 GP learning repetitions for a given configuration. In rows 1–20, the numbers of solutions with the fitness function value included in the indicated decade are specified. The table also provides the numbers of solutions better than the minimum obtained by the PID (row 21), exact mean values (row 22), and exact minimum values (row 23) of the fitness function.

The data in Figure 7 and Table 4 reveal that it is actually statistically difficult for the GP learning to discover a control algorithm which suppresses the stick-slip better than the PID. The mean GP result is worse than PID for every configuration, and the percentage of GP individuals better than PID varies from 2 for NAVC03 to 38 for NA02. On the other hand, the 100 repetitions suffice to find very well-fitted individuals with the fitness function value many order of magnitude lower than the one obtained by the PID algorithm. The learning is most effective in the case of the full signal configurations and the configurations without only the acceleration feedback. Other configurations, commonly characterized by the absence of velocity feedback, result in worse fitness of the best individuals. This is somewhat counterintuitive, as one could expect that acceleration may be the important feedback signal, giving direct information about the actual state of the controlled system. The results also reveal that the variation of mutation probability in the range [0.1,0.3] has no systematic impact on the learning efficiency. Therefore, no concrete probability value is preferred, but one can use the probability variation to diversify the GP learning process.

**Figure 8:**
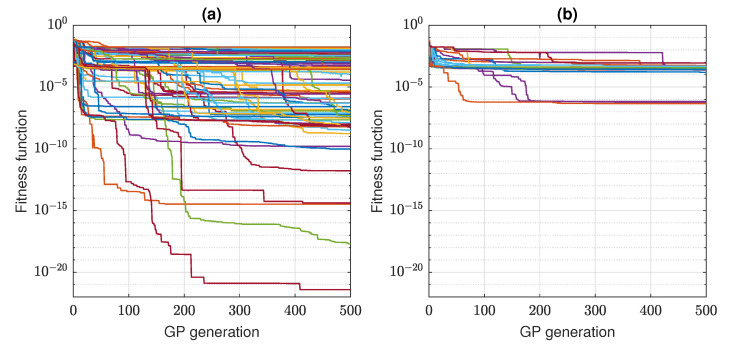
GP learning progress: (**a**) NA02; (**b**) NAVC02.

In Figure 8, the progress of GP learning is shown in terms of the fitness function value obtained by the best individual in consecutive generations. There is a plot for each of the 100 repetitions. The configurations NA02 (a) and NAVC02 (b) are compared, including the globally best and worst individuals, respectively. There is an evident difference between these configurations, and the data provided to a discovered algorithm in the variant NAVC02 (without $a^{*}$, $v^{*}$, $u^{*}$) is clearly too limited for effective learning. The plots in Figure 8a indicate that many repetitions of the evolutionary process were not stagnated at the moment of their termination after 500 generations, and they could improve solutions if they run longer.

**Figure 9:**
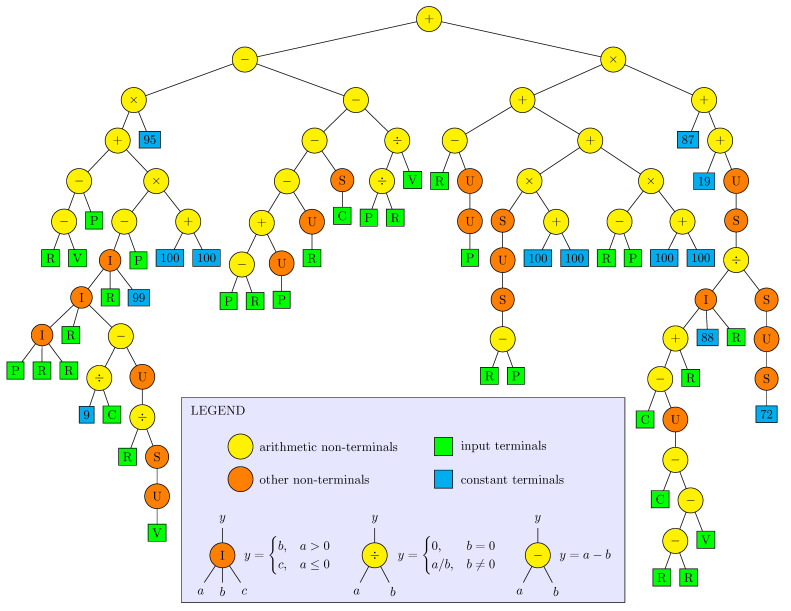
Parse-tree of the best individual.

So far, the GP learning results have been analyzed statistically. Let us now study closer the best obtained individuals. The globally best algorithm has been found for the configuration NA02; and its fitness function value is $3.94\times 10^{-22}$. Its parse-tree is presented in Figure 9. The tree seems to be too large and complicated to interpret the algorithm it represents. One can, however, observe a few specific details. The sub-expression R−P or P−R, representing actually a position error, is present in the tree a few times. This is an elementary expression expected in the algorithm of position controller. There are three occurrences of the sub-expression 100+100, which obviously evaluates to 200. Presumably, the evolutionary process constructed this sub-expression once and then multiplied it crossing individuals, finding the constant 200 useful for a well-fitted individual, while a single terminal can represent an integer constant in the interval [1,100]. There are “dead” sub-expressions in the parse-tree, which is quite common for products of evolutionary algorithms, as they cannot detect that some part of genetic information is trivial or redundant. In particular, the sub-expression I(P,R,R) evaluates to R, and the sub-expression R−R evaluates to 0.

**Figure 10:**
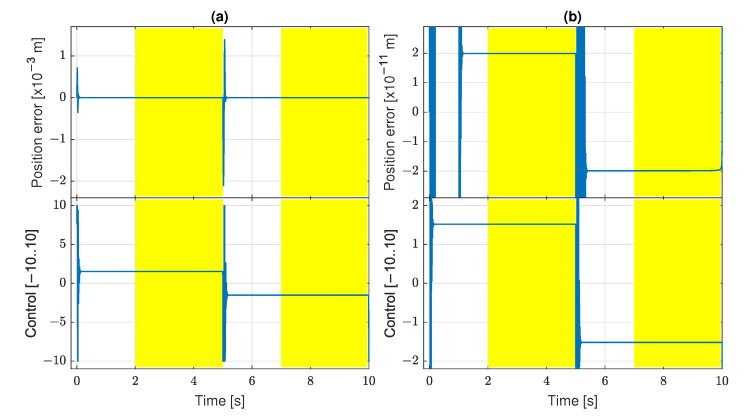
Tracking quality for best individual: (**a**) full range of signal values; (**b**) signal values in steady state.

In Figure 10, the tracking quality for the best individual is presented in the form of the position error, i.e., $p_{\mathrm{ref}}^{*}-p^{*}$, and the control signal. In part (a), the signals are presented in the full range of their variability. In part (b), the regions taken into account for calculation of the fitness function value are zoomed. There are two important observations. First, the penalty used for elimination of control signal oscillations works properly. The control signal has bang-bang oscillations only at short transient phases around the 0 and 5 s, but it keeps a constant value during the established ramp signal tracking. This constant control signal counteracts the friction force. Secondly, during the ramp signal tracking, the position error settles on the value about $\pm 2\times 10^{-11}\;m$, which is actually immeasurable. Therefore, the best individual and many others with a similar value of the fitness function represent control algorithms that suppress the stick-slip practically ideally.

**Figure 11:**
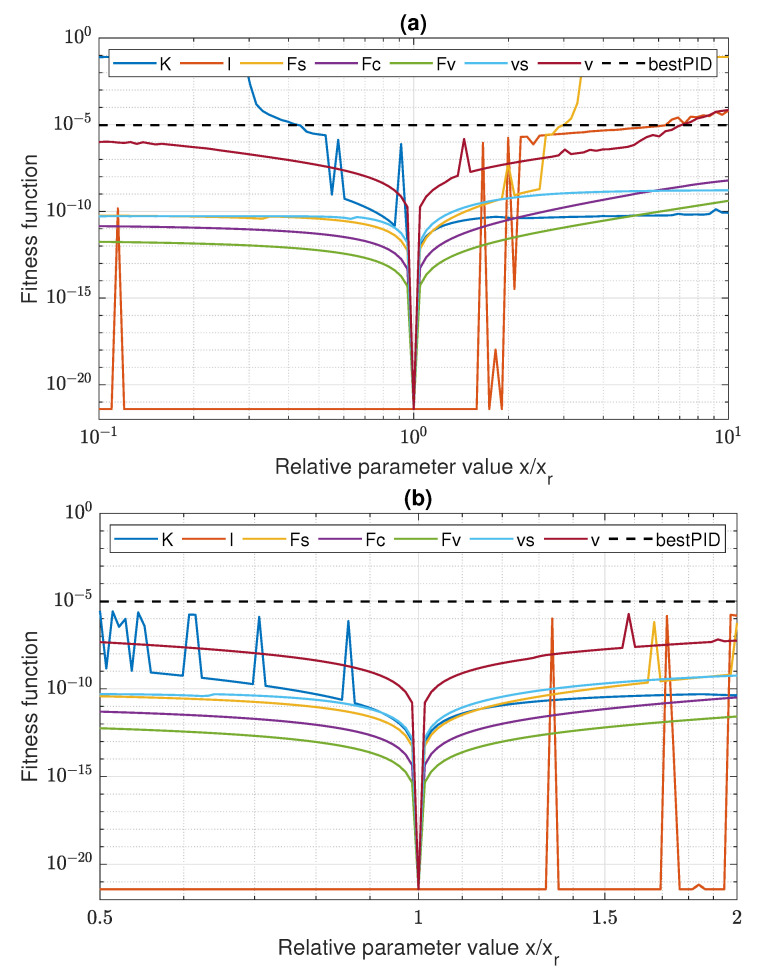
Servo control sensitivity: (**a**) $x\in [0.1x_{r},10x_{r}]$; (**b**) $x\in [0.5x_{r},2x_{r}]$.

The ideal position tracking presented in Figure 10 may be a result of overfitting, and there is a risk of quality deterioration when the system parameters are changed. To verify this, the sensitivity of the fitness function value of the best individual on the parameter deviations has been analyzed. For each parameter $x\in \{K,I,F_{s},F_{c},F_{v},v_{s},v\}$, the fitness function value has been computed in the interval $\left[0.1x_{r},10x_{r}\right]$, where $x_{r}$ is the reference value of *x* used in the GP training. In particular, the parameter *v* represents the slope of the reference trajectory, which deviates from its training value 0.1, indicated in (3) and Figure 4a. The results are shown in the full scope in Figure 11a and zoomed in the interval $\left[0.5x_{r},2x_{r}\right]$ in Figure 11b. The plots indicate that the fitness function value becomes exceptionally low only very close to the training parameter values. However, the algorithm fitness remains relatively good and much better than the PID fitness in broad intervals of parameter values.

**Figure 12:**
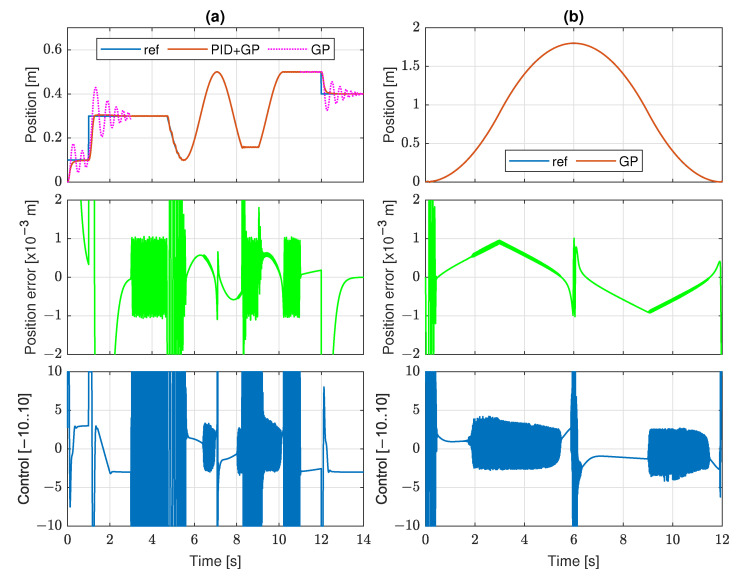
Tracking quality of composite trajectories: (**a**) piecewise constant and sinusoidal; (**b**) parabolic.

The sensitivity analysis exhibits that the algorithm can suppress stick-slip for a wide range of the reference velocities. However, the analysis has been performed using the trajectory of the shape shown in Figure 4a with a piecewise constant velocity, and it is not clear whether the performance is also satisfactory for a variable velocity. Therefore, additional tests have been carried out to verify the algorithm for more complex trajectories, in particular, trajectories with a smoothly changing velocity. The trajectories are shown in Figure 12. The first trajectory (Figure 12a) consists of parts with sinusoidal shapes, as well as constant position intervals; it also includes two steps at time 1 and 12 s. The second one (Figure 12b) is a piecewise parabolic continuous trajectory. The plots reveal that the algorithm has a good performance in tracking of non-constant smooth parts of the trajectories, even if the motion has a variable velocity. In such cases, the stick-slip is usually suppressed to an irrelevant level, but an exception can be found for the first decreasing part of the sinusoidal shape. On the other hand, the GP discovered algorithm copes poorly with the constant reference position and steps. It is not surprising, as these are conditions for which the algorithm was not trained. If switching the GP algorithm to the standard PID in the time intervals [0,3] and [11,14], the steps are handled much better and the control signal is smooth (line PID+GP). The switching itself does not disturb the tracking.

**Figure 13:**
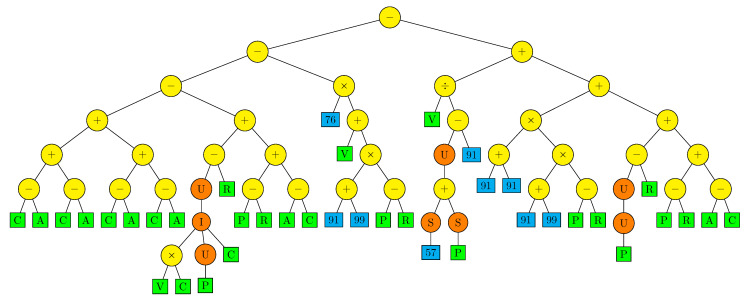
Parse-tree of the second best individual.

It is also interesting to see the parse-tree of the second globally best solution (Figure 13). Its fitness function value is $1.03\times 10^{-21}$ and the difference with the best one is practically insignificant. This algorithm has been obtained for the configuration F02, so it has the access to acceleration feedback. The parse-tree reveals that the algorithm extensively uses this feedback. In particular, the acceleration terminal A is almost in any case in the sub-expression C−A or A−C representing the acceleration error because K=I=1, hence a≅u. Therefore, similarly as for the position feedback, the acceleration feedback is employed in the form that one could expect in a reasonably designed control algorithm. It is intriguing that, having the acceleration feedback accessible, the GP process has used it in a natural way, but it also found a similarly well-fitted individual without it.

**Figure 14:**
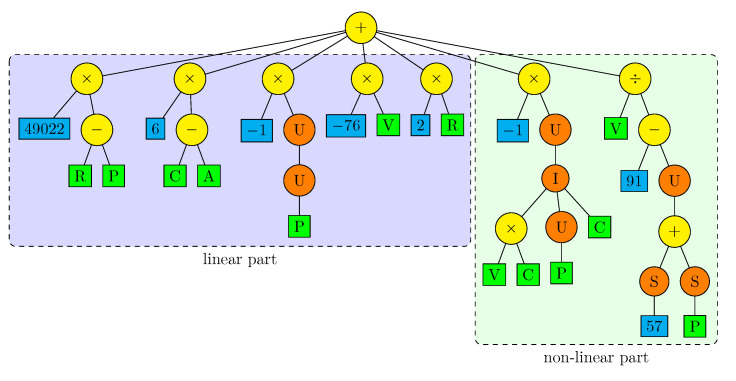
Simplified and ordered parse-tree of second best individual.

The second best individual turned out to be simpler than the best one because many parts of its parse-tree can be algebraically simplified and ordered. It is also easy to split it into a linear and nonlinear parts. The resultant equivalent parse-tree is presented in Figure 14. The nonlinear part is particularly tiny. A direct analysis and interpretation of this algorithm would probably be possible.

### 3.3. Control Algorithms for Servo with Real Position Sensor

Let us consider now a realistic imperfect measurement. We assume the usual implementation of a real servo, in which there is only a position sensor in the form of a quadrature encoder. Such a sensor quantizes actual position value. Let the quantization step be equal to 0.1mm, then

(8) $$P_{k}=p^{*}\left(k\Delta^{*}\right)=\frac{\left\lfloor 10,000\;p(k\Delta^{*})\right\rfloor }{10,000}.$$

The velocity and acceleration signals are not directly measured. They need to be estimated numerically. Two scenarios have been considered, being alternative implementations of the conditioner block (Figure 2). In the first implementation (*a*-scenario), the velocity and acceleration are obtained in the simplest way, by calculation of the differences

(9) $$V_{k}=\frac{P_{k}-P_{k-1}}{\Delta^{*}},\;A_{k}=\frac{V_{k}-V_{k-1}}{\Delta^{*}},\;P_{0}=V_{0}=0.$$

It is well-known that such calculation degrades signal quality by amplification of quantization noise. For this reason, a real/filtered form of differentiation is used, e.g., in derivative blocks of PID controllers. More advanced solutions have also been proposed [35]. In the considered second scenario (*b*-scenario), the standard filtered derivative is used. It has the continuous-time transfer function $s/(T_{f}s+1)$, and the ZOH-equivalent discrete-time form $\left(z-1\right)/\left(T_{f}z-T_{f}p_{f}\right)$, which implies the following recursive formulas:

(10) $$V_{k}=\frac{P_{k}-P_{k-1}}{T_{f}}+p_{f}V_{k-1},\;A_{k}=\frac{V_{k}-V_{k-1}}{T_{f}}+p_{f}A_{k-1},\;P_{0}=V_{0}=A_{0}=0,$$

where the settings $T_{f}=0.002\;s$ and $p_{f}=\exp\left(-\Delta^{*}/T_{f}\right)=0.607$ have been applied for the filter time constant and its pole, respectively.

**Figure 15:**
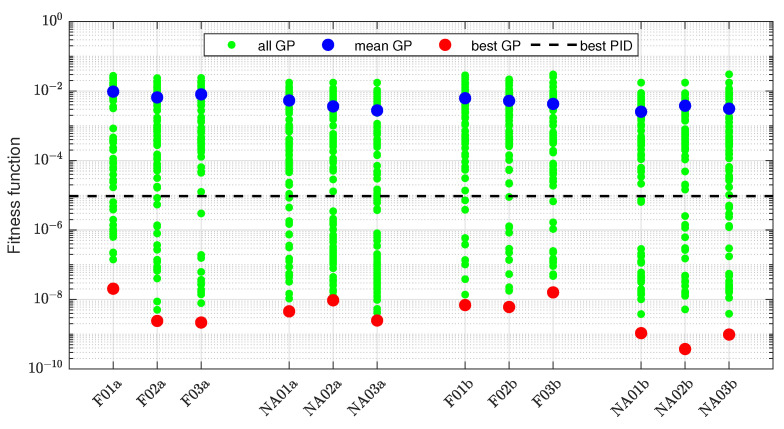
Comparison of GP learning results for servo with real position sensor.

Experiments have been conducted only for the configurations including the velocity feedback (F01-3, NA01-3), assuming that it is required for good results, as observed in the previous case of the ideal measurement. The obtained distribution of the fitness function value is presented in Figure 15. It is evident that the best individuals are now remarkably worse than for the ideal measurement. On the other hand, there are still many individuals significantly better than the best PID-based case. One can also observe that different configurations, measurement scenarios, and mutation levels have little impact on the results.

More details are provided in Table 5. Compared to the data in Table 4, the subset of the individuals with $J\in [10^{-22},10^{-10}]$ is empty. However, for the greater values of the fitness function, the distribution is favorable. There are relatively many solutions with $J\in [10^{-9},10^{-7}]$. The number of results outperforming the PID is also relatively large, e.g., it is 46 for NA03a, better than in all the experiments for ideal measurement. It means that it is harder to find extremely well-fitted individuals when the measurement is realistic, but it is not so hard to find sufficiently good individuals. According to the row 10 in Table 5, the configuration without acceleration feedback increases a chance to obtain a result better than the PID-based minimum. The globally best individuals have been discovered for the configuration without acceleration feedback and with filtered derivatives (NA01-3b).

**Figure 16:**
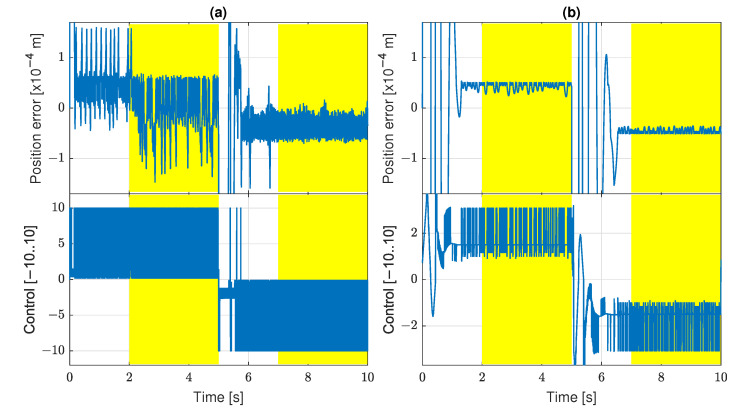
Tracking quality for scenarios (**a**,**b**) with real sensors.

The tracking quality is illustrated in Figure 16 for the best individuals of the scenarios *a* and *b*. The problem of oscillations reappears. The control signal oscillates without changing the sign, so the individual is not discarded by the zero-crossing penalty. It indicates that it is harder or impossible for the GP to discover a non-oscillating control in the case of real measurement. There are full-scale bang-bang unipolar oscillations in the scenario *a*. The oscillations are significantly reduced in the scenario *b* in which filtered derivatives are applied for estimation of velocity and acceleration.

## 4. Discussion

One can observe that discovering of a good algorithm for servo stick-slip suppression is a quite difficult task for the GP. A relatively small part of the GP training attempts results in a fitness function value better than the best one obtained by the plain PID control, even though the number of generations is large. Indeed, the number of generations amounts to 500 here (Table 2) while, e.g., a training based on not more than 20 generations was sufficient to discover successful strategies for robot RealTimeBattle [26]. However, the large number of generations is really required in this experiments, as the generations evolve slowly and often do not stagnate even after the 500 stages (Figure 8). Despite these difficulties, the GP evolutionary process has found many well-fitted individuals that proves the existence of algorithms effectively suppressing stick-slip. These algorithms are represented by quite simple, but obviously nonlinear expressions. It is hard to expect an effective compensation of the nonlinear friction force by a linear control rule.

The fitness function values are actually unrealistically low for the variant of ideal measurement. Switching to the real measurement scenarios makes a remarkable difference. It shows how important the measurement in such precise nonlinear control is, and that details of sensors characteristic and signal conditioning rules should be included in the model to obtain reliable results. It is a symptomatic discovery of the GP that the acceleration feedback is not needed for a good control algorithm, but the tachometric feedback is important. While some studies claim that the acceleration feedback improves servo control quality, it may not be the case for the stick-slip suppression or it may depend on some unrecognized details of a servo system model.

The results confirm the general usefulness of the GP for discovering friction-resistant servo control algorithms. The results also provide some hints on how to improve the approach in the future. While the zero-crossing penalty has prevented bipolar bang-bang oscillations of the control signal, it has not eliminated unipolar oscillations observed in Figure 16. To achieve better suppression, a more restrictive penalty is needed with more sensitive oscillation detection. It is also a disadvantage that the GP cannot capture the symmetry between positive and negative slopes of the reference trajectory, as one can observe in Figure 6 and on the examples of all the presented parse-trees (Figure 9, Figure 13 and Figure 14), which do not represent functions symmetric with respect to sign of V. For a practical application, one can extract only the part of the function for one sign of velocity and symmetrize it directly, but it would be more advantageous to restrict the GP, so that it does not produce the asymmetric control algorithms. Finally, while the obtained algorithms are essentially immune to parameter changes (Figure 11), one would expect lower sensitivity on the actual reference velocity *v*, as this parameter can vary in a broad range in a real system. It seems that the individuals obtained in this work are overfitted for the slope value used in the learning reference trajectory. To improve the algorithms in this aspect, a more diversified reference trajectory may be used, having a variable velocity or components of different slope values.

## 5. Conclusions

Although it seems to be impossible to apply the GP for learning servo stick-slip compensation in real-time, the discovered well-fitted control algorithms can be implemented in a real-world servo system after the off-line training. Modern embedded servo controllers are fast enough to cyclically execute calculation described by the obtained parse-trees. Therefore, the approach can be useful in practice. A practical use has to be preceded by identification of real system parameters, and then the GP learning may be conducted offline using simulation. The result of this work indicates that the learning outcome strongly depends on details of the modeled and simulated system; in particular, it depends on the selected sensors set, sensors accuracy, and signal conditioning. One can notice that the GP method can also be applied to the emerged problem of identification of real servo system parameters. It will provide a non-parametric model which may be especially advantageous for the modeling of friction, as friction does not have a unique well-established parametric model. The experiments on a real system remain the subject of future work.

## Figures and Tables

**Table 1:** GP node functions.

Symbol	Arity	Operation	Explanation
+	2	arithmetic addition	
−	2	arithmetic subtraction	
*	2	arithmetic multiplication	
÷	2	safe arithmetic division	÷(a,b)=ifb=0then0elsea/bend
I	3	if-else expression	I(a,b,c)=ifa>0thenbelsecend
S	1	sin function	S(a)=sina
U	1	unit delay	
{1,…,100}	0	integer constant	randomly initialized and mutated
R	0	reference position	from trajectory generator
C	0	control signal	from previous control cycle
P	0	actual position	from position sensor
V	0	actual velocity	from velocity sensor (or estimator)
A	0	actual acceleration	from acceleration sensor (or estimator)

**Table 2:** Basic configuration of GP.

Parameter	Setting
Initial population	ramped half-and-half (2:6) algorithm
Breeding pipelines	reproduction, crossover, mutation
Reproduction probability	0.1
Crossover probability	0.9
Mutation probability	0.1, 0.2, 0.3
Selection method	tournament without elitism
Tournament size	7
Size of population	1000
Number of generations	500

**Table 3:** GP experiment configurations.

Mutation Probability	Full Input	No Acc	No Vel	No Acc, No Vel	No Acc, No Vel, No Ctr
**0.1**	F01	NA01	NV01	NAV01	NAVC01
**0.2**	F02	NA02	NV02	NAV02	NAVC02
**0.3**	F03	NA03	NV03	NAV03	NAVC03

**Table 4:** GP learning results for ideal sensors.

No.	Parameter	F01	F02	F03	NA01	NA02	NA03	NV01	NV02	NV03	NAV01	NAV02	NAV03	NAVC01	NAVC02	NAVC03
1	$\#\left\{s\in S:J\left(s\right)\in \left[10^{-2},10^{-1}\right]\right\}$	44	30	30	28	19	13	16	31	22	5	9	5			
2	$\#\left\{s\in S:J\left(s\right)\in \left[10^{-3},10^{-2}\right)\right\}$	27	29	30	27	24	31	24	16	20	14	11	11	4		2
3	$\#\left\{s\in S:J\left(s\right)\in \left[10^{-4},10^{-3}\right)\right\}$	13	18	15	19	14	15	32	28	29	66	68	63	93	97	94
4	$\#\left\{s\in S:J\left(s\right)\in \left[10^{-5},10^{-4}\right)\right\}$	2	5	1	2	5	8	12	7	9	6	4	4			2
5	$\#\left\{s\in S:J\left(s\right)\in \left[10^{-6},10^{-5}\right)\right\}$	2	2	3	1	7	6	5	9	6	5	5	11	1		1
6	$\#\left\{s\in S:J\left(s\right)\in \left[10^{-7},10^{-6}\right)\right\}$	2	3	6	5	7	3	9	5	6	4	1	2	2	3	1
7	$\#\left\{s\in S:J\left(s\right)\in \left[10^{-8},10^{-7}\right)\right\}$	6	6	8	7	9	12	1	1	2			2			
8	$\#\left\{s\in S:J\left(s\right)\in \left[10^{-9},10^{-8}\right)\right\}$	1	1	1	3	8	7	1		2		2				
9	$\#\left\{s\in S:J\left(s\right)\in \left[10^{-10},10^{-9}\right)\right\}$		1	1	1	1	2		1	2			2			
10	$\#\left\{s\in S:J\left(s\right)\in \left[10^{-11},10^{-10}\right)\right\}$		2		2	1	1		1	1						
11	$\#\left\{s\in S:J\left(s\right)\in \left[10^{-12},10^{-11}\right)\right\}$	1		1	1	1										
12	$\#\left\{s\in S:J\left(s\right)\in \left[10^{-13},10^{-12}\right)\right\}$	1														
13	$\#\left\{s\in S:J\left(s\right)\in \left[10^{-14},10^{-13}\right)\right\}$				2				1							
14	$\#\left\{s\in S:J\left(s\right)\in \left[10^{-15},10^{-14}\right)\right\}$			1		2										
15	$\#\left\{s\in S:J\left(s\right)\in \left[10^{-16},10^{-15}\right)\right\}$		1	1	1											
16	$\#\left\{s\in S:J\left(s\right)\in \left[10^{-17},10^{-16}\right)\right\}$	1								1						
17	$\#\left\{s\in S:J\left(s\right)\in \left[10^{-18},10^{-17}\right)\right\}$			1	1	1										
18	$\#\left\{s\in S:J\left(s\right)\in \left[10^{-19},10^{-18}\right)\right\}$		1	1			2									
19	$\#\left\{s\in S:J\left(s\right)\in \left[10^{-21},10^{-20}\right)\right\}$		1													
20	$\#\left\{s\in S:J\left(s\right)\in \left[10^{-22},10^{-21}\right)\right\}$					1										
21	$\#\left\{s\in S:J\left(s\right)<J_{\mathrm{minPID}}\right\}$	14	18	24	24	38	33	16	18	20	9	8	17	3	3	2
22	${\mathrm{mean}}_{s\in S}J\left(s\right)\;\left[\times 10^{-4}\right]$	85.5	65.3	61.3	61.1	39.2	34.0	38.0	57.1	45.4	16.0	23.2	14.2	4.72	3.43	3.78
23	$\min_{s\in S}J\left(s\right)$	$8.22\;\times \;10^{-17}\;\;$	$1.03\;\times \;10^{-21}\;\;$	$4.18\;\times \;10^{-19}\;\;$	$1.08\;\times \;10^{-18}\;\;$	$3.94\;\times \;10^{-22}\;\;$	$1.30\;\times \;10^{-19}\;\;$	$6.03\;\times \;10^{-9}\;\;$	$3.60\;\times \;10^{-14}\;\;$	$1.68\;\times \;10^{-17}\;\;$	$2.87\;\times \;10^{-7}\;\;$	$1.05\;\times \;10^{-9}\;\;$	$2.12\;\times \;10^{-10}\;\;$	$2.35\;\times \;10^{-7}\;\;$	$4.60\;\times \;10^{-7}\;\;$	$1.03\;\times \;10^{-7}\;\;$

**Table 5:** GP learning results for servo with real position sensors.

No.	Parameter	F01a	F02a	F03a	NA01a	NA02a	NA03a	F01b	F02b	F03b	NA01b	NA02b	NA03b
1	$\#\left\{s\in S:J\left(s\right)\in \left[10^{-2},10^{-1}\right]\right\}$	48	32	43	24	14	9	24	17	5	5	11	8
2	$\#\left\{s\in S:J\left(s\right)\in \left[10^{-3},10^{-2}\right)\right\}$	15	21	14	22	30	20	41	41	56	33	38	32
3	$\#\left\{s\in S:J\left(s\right)\in \left[10^{-4}10^{-3},\right)\right\}$	15	24	24	23	16	17	21	25	15	31	25	21
4	$\#\left\{s\in S:J\left(s\right)\in \left[10^{-5},10^{-4}\right)\right\}$	8	7	4	10	7	8	5	5	10	8	5	11
5	$\#\left\{s\in S:J\left(s\right)\in \left[10^{-6},10^{-5}\right)\right\}$	6	4	1	4	5	7	2	3	3	3	4	7
6	$\#\left\{s\in S:J\left(s\right)\in \left[10^{-7},10^{-6}\right)\right\}$	7	5	2	7	17	12	4	5	5	7	5	2
7	$\#\left\{s\in S:J\left(s\right)\in \left[10^{-8},10^{-7}\right)\right\}$	1	4	10	9	9	23	2	3	6	11	10	17
8	$\#\left\{s\in S:J\left(s\right)\in \left[10^{-9},10^{-8}\right)\right\}$		3	2	1	2	4	1	1		2	1	1
9	$\#\left\{s\in S:J\left(s\right)\in \left[10^{-10},10^{-9}\right)\right\}$											1	1
10	$\#\left\{s\in S:J\left(s\right)<J_{\mathrm{minPID}}\right\}$	14	16	15	21	33	46	9	12	14	23	21	28
11	${\mathrm{mean}}_{s\in S}J\left(s\right)\;\left[\times 10^{-4}\right]$	96.0	65.5	80.2	53.5	36.2	27.7	62.1	52.3	42.5	25.4	37.7	31.1
12	$\min_{s\in S}J\left(s\right)$	$2.04\;\times \;10^{-8}\;\;$	$2.42\;\times \;10^{-9}\;\;$	$2.17\;\times \;10^{-9}\;\;$	$4.56\;\times \;10^{-9}\;\;$	$9.49\;\times \;10^{-9}\;\;$	$2.48\;\times \;10^{-9}\;\;$	$6.90\;\times \;10^{-9}\;\;$	$6.09\;\times \;10^{-9}\;\;$	$1.60\;\times \;10^{-8}\;\;$	$1.07\;\times \;10^{-9}\;\;$	$3.75\;\times \;10^{-10}\;\;$	$9.78\;\times \;10^{-10}\;\;$

## Data Availability

The data supporting results consist of the GP learning system source code (Java) and output files generated by the learning system that include parse-trees and fitness function values of all the evaluated individuals for all configurations and repetitions.

## Abbreviations

The following abbreviations are used in this manuscript:

GP	Genetic Programming
ECJ	Evolutionary Computation [research system written in] Java
